# Mifepristone prevents repopulation of ovarian cancer cells escaping cisplatin-paclitaxel therapy

**DOI:** 10.1186/1471-2407-12-200

**Published:** 2012-06-22

**Authors:** Alicia A Goyeneche, Carlos M Telleria

**Affiliations:** 1 Division of Basic Biomedical Sciences, Sanford School of Medicine of The University of South Dakota, 414 East Clark Street, Vermillion, SD, USA; 2 Present address: Institute of Histology and Embryology of Cuyo, National Council for Scientific and Technical Research (CONICET), Mendoza, Argentina; 3 Present address: Institute of Medicine and Experimental Biology of Cuyo, National Council for Scientific and Technical Research (CONICET), Mendoza, Argentina

## Abstract

**Background:**

Advanced ovarian cancer is treated with cytoreductive surgery and combination platinum- and taxane-based chemotherapy. Although most patients have acute clinical response to this strategy, the disease ultimately recurs. In this work we questioned whether the synthetic steroid mifepristone, which as monotherapy inhibits the growth of ovarian cancer cells, is capable of preventing repopulation of ovarian cancer cells if given after a round of lethal cisplatin-paclitaxel combination treatment.

**Methods:**

We established an *in vitro* approach wherein ovarian cancer cells with various sensitivities to cisplatin or paclitaxel were exposed to a round of lethal doses of cisplatin for 1 h plus paclitaxel for 3 h. Thereafter, cells were maintained in media with or without mifepristone, and short- and long-term cytotoxicity was assessed.

**Results:**

Four days after treatment the lethality of cisplatin-paclitaxel was evidenced by reduced number of cells, increased hypodiploid DNA content, morphological features of apoptosis, DNA fragmentation, and cleavage of caspase-3, and of its downstream substrate PARP. Short-term presence of mifepristone either enhanced or did not modify such acute lethality. Seven days after receiving cisplatin-paclitaxel, cultures showed signs of relapse with escaping colonies that repopulated the plate in a time-dependent manner. Conversely, cultures exposed to cisplatin-paclitaxel followed by mifepristone not only did not display signs of repopulation following initial chemotherapy, but they also had their clonogenic capacity drastically reduced when compared to cells repopulating after cisplatin-paclitaxel.

**Conclusions:**

Cytostatic concentrations of mifepristone after exposure to lethal doses of cisplatin and paclitaxel in combination blocks repopulation of remnant cells surviving and escaping the cytotoxic drugs.

## Background

Ovarian cancer is the most lethal gynecologic disease [1]. Because early detection biomarkers are not yet available and the symptomatology is vague, the disease is usually diagnosed at a late stage when growths have extended within the peritoneal cavity [2-4]. At this point, patients usually undergo cytoreductive surgery followed by platinum plus taxane-based chemotherapy [1,3]. The response to this regime is successful with disease remission in at least 70% of the cases; however, the majority of first responders will relapse within 18 months with a platinum-resistant disease [3-6]. Unfortunately, there is no current agreed maintenance therapy following the initial cisplatin-paclitaxel chemotherapy regimen [5,6], and the median survival time for patients after recurrence is only approximately two years [7].

Cisplatin was adopted as primary chemotherapy schedule in the 1970s in association with cyclophosphamide [5]. In the 1990s a microtubule stabilizer, paclitaxel, was shown to potentiate cisplatin-based therapy in ovarian cancer patients with better efficacy than cisplatin-cyclophosphamide [8,9]. Since these clinical trials, only minor variations in the standard chemotherapeutic schedule for ovarian cancer patients have been implemented. For instance, in the early 2000s it was demonstrated that carboplatin was equality effective as cisplatin in association with paclitaxel but with much less nephrotoxicity, and better tolerability and quality of life than cisplatin [10]. Thus, cisplatin and paclitaxel, and later carboplatin plus paclitaxel, have been broadly accepted as first-line chemotherapy for advanced epithelial ovarian cancer. Another improvement in overall survival was achieved by the adoption of intraperitoneal delivery of these drugs when compared with intravenous administration [11]. However, data worldwide concur that in the past 20 years there has been little change in the 5-year survival rates post-diagnosis of patients with ovarian cancer [1].

Our laboratory provided evidence that the synthetic steroid mifepristone is effective as a single agent *in vitro* and *in vivo* blocking the growth of human epithelial ovarian cancer cells [12]. When used at concentrations likely to be achieved *in vivo* in humans [13-16], mifepristone inhibited cell growth by inducing G1 cell cycle arrest associated with inhibition of DNA synthesis, downregulation of the transcription factor E2F1 needed for S phase progression, and inhibition of the activity of cyclin dependent kinase 2 [12,17], which is critical to promote G1 to S phase transition [18]. We also reported that the growth inhibitory effect of mifepristone in ovarian cancer cells does not require the expression of cognate progesterone receptors [19], and is independent of p53 functionality and platinum sensitivity [20], making mifepristone an even more interesting chemotherapeutic candidate for ovarian cancer as the majority of tumors in relapsing patients are platinum resistant and p53 mutant [7]. Finally, we have shown in ovarian cancer cells that mifepristone potentiates the lethality of otherwise sub-lethal doses of cisplatin, and synergizes with cisplatin growth inhibiting ovarian cancer cells of different genetic backgrounds and platinum sensitivities [21].

In this work we set out to study whether mifepristone has the capacity to block repopulation or regrowth of ovarian cancer cells escaping front-line cisplatin plus paclitaxel chemotherapy. We report that although ovarian cancer cells were initially severely damaged by cisplatin-paclitaxel, the cultures eventually recovered due to the proliferation of escape cells. Such cell repopulation, nonetheless, was blunted by the chronic presence of clinically relevant doses of mifepristone.

## Methods

### Cell lines, culture conditions and treatments

The human ovarian carcinoma cell lines OV2008, A2780, and IGROV-1 were obtained in 2003 from Dr. Stephen Howell (University of California, San Diego) [22]. The cells were maintained in RPMI 1640 (Mediatech, Herndon, VA) supplemented with 5% or 10% (OV2008 or A2780/IGROV-1, respectively) heat inactivated FBS (Atlanta Biologicals, Lawrencenville, GA), 10 mM HEPES (Mediatech), 4 mM L-glutamine (Mediatech), 1 mM sodium pyruvate (Mediatech), 100 IU penicillin (Mediatech), and 100 μg/ml streptomycin (Mediatech). SK-OV-3 ovarian cancer cells were obtained from the American Type Culture Collection (ATCC, Manassas, VA) and were routinely maintained in RPMI 1640 (Mediatech) supplemented with 10% FBS (Atlanta Biologicals), 10 mM HEPES (Mediatech), 4 mM L-glutamine (Mediatech), 0.45% D (+) glucose (Sigma Chemical Company, St. Louis, MO), 1 mM sodium pyruvate (Mediatech), 1 X non-essential amino acids (Mediatech), 100 IU penicillin (Mediatech), 100 μg/ml streptomycin (Mediatech), and 0.01 mg/ml human insulin (Roche, Indianapolis, IN) as we previously described [12,17,19,20]. All cell lines were cultured at 37°C in a humidified atmosphere in the presence of 5% CO_2_.

The stock of cisplatin (cis-diamminedichloroplatinum II) (cisplatin; Sigma) was a 3 mM solution in 0.9% NaCl. Cells were exposed to cisplatin for only 1 h; thereafter, the medium was replaced with fresh cisplatin-free medium. The stock of paclitaxel (Sigma) was a 100 μM solution in DMSO. Cells were exposed to paclitaxel for 3 h; thereafter, the medium was replaced with fresh paclitaxel-free medium. Treatment of cells with mifepristone (Sigma) was done from a 20 mM stock solution in DMSO, which was maintained at −20°C. The maximal concentration of DMSO in medium was less than 0.02% (v/v).

### Cell proliferation

Triplicate cultures were trypsinized, pelleted by centrifugation at 500 *g* for 5 min, and washed with PBS. The cells were resuspended in ViaCount reagent (Guava Technologies, Hayward, CA) and studied using the Guava ViaCount application in the Guava EasyCyte Mini microcapillary cytometer (Guava Technologies) as we previously reported [20]. For data presentation purposes, when ‘growth’ is indicated, controls are considered 100%, whereas when ‘relative growth’ is stated, the number of cells at the beginning of the experiment was considered as 1.

### Phase contrast microscopy

Along the various treatment paradigms, cells maintained in 6-well plates where observed and photographed using a Zeiss Axiovert M200 inverted microscope with a phase contrast objective (Carl Zeiss, Thornwood, NY).

### Determination of sub-G1 DNA content and cell cycle stages

After treatment, cells were trypsinized, pelleted by centrifugation at 500 *g* for 5 min, washed with PBS, and fixed with 4% paraformaldehyde. Cells were once again washed with PBS and pelleted by centrifugation at 500 *g* for 5 min. Then, approximately 100,000–200,000 cells were resuspended in 200 μl of cell cycle buffer [3.8 mM sodium citrate (Sigma), 7 U/ml RNase A (Sigma), 0.1% (v/v) Triton X-100 (Sigma), and 0.05 mg/ml propidium iodide (Sigma)], at a concentration of 500–1000 cells/μl. Cells were studied for the capacity of their DNA to bind propidium iodide utilizing the Guava EasyCyte Mini microcapillary cytometer and the cell cycle application of the CytoSoft 4.1 software (Guava Technologies). In some circumstances, to calculate changes in hypodiploid DNA content induced by treatment, we expressed the data as specific sub-G1 as follows: specific sub-G1 = [100* (treated sub-G1 – control sub-G1)/(100 – control sub-G1)].

### DNA fragmentation

Floating and adherent cells were pelleted and digested overnight at 50°C in a buffer composed of 100 mM NaCl, 10 mM Tris HCl (pH 8.0), 25 mM EDTA (pH 8.0), 0.5% SDS and 0.1 mg/ml proteinase K (Life Technologies, Rockville, MD). The genomic DNA was extracted from the digested cells with phenol/chloroform/isoamyl alcohol (25:24:1, v/v/v), precipitated, and digested for 60 min at 37°C with 1 μg/ml ribonuclease (deoxyribonuclease-free; Roche, Indianapolis, IN). After extraction and precipitation, an equal amount of DNA for each sample (2 μg) was separated by electrophoresis on a 2.5% agarose gel, impregnated with SYBR Gold nucleic acid gel stain (Molecular Probes, Eugene, OR) and photographed with the Amersham Typhoon Fluorescence imaging system (Amersham Biosciences Corp., Piscataway, NJ). A 100 bp DNA ladder (Promega, Madison, WI) was utilized to determine the size of the fragments of DNA.

### SDS-PAGE and western blotting

Cells were scraped, pelleted, washed twice with PBS, and lysed by the addition of two volumes of radioimmunoprecipitation assay buffer (RIPA) containing 50 mM Tris- HCl (pH 7.4), 150 mM NaCl, 1% NP-40 (Sigma), 0.25% sodium deoxycholate (Sigma), 1 mM EDTA, 1 mM PMSF (Sigma), 1 μg/ml pepstatin (Sigma), 1 mM orthovanadate (Sigma) and 1 mM sodium fluoride (Sigma). Cells were disrupted by passing them through a 21 gauge needle, and gently rocked on ice for 30 min. Lysates were centrifuged at 16,000 *g* for 15 min at 4°C, and the supernatant was considered the whole cell extract, which was assayed for protein content by using the bicinchoninic acid method (BCA; Pierce, Rockford, IL). Equivalent amounts of protein (50 μg) per point were loaded in 12% (w/v) acrylamide gels, subjected to SDS-PAGE and transferred to PVDF membranes. The blots were blocked in 5% (v/v) nonfat milk in TBS containing 0.1% (v/v) Tween 20 (T). Blots were then probed overnight with primary antibodies against poly (ADP-ribose) polymerase (PARP) (#9542; 1:1000; Cell Signaling Technologies, Danvers, MA) or caspase caspase-3 (#9662; 1:1000; Cell Signaling). The membranes were washed 3 × 5 min in TBS-T and incubated with 1:10,000 dilution of peroxidase-conjugate secondary antibody (#111-035-003; Jackson ImmunoResearch Laboratories, West Grove, PA) for 30 min at room temperature. The blots were again washed, developed by chemiluminescence, and exposed to radiographic film. Blots were also probed with an antibody directed against glyceraldehyde-3-phosphate dehydrogenase (GAPDH) (ab9485; 1:10,000; Abcam, Cambridge, MA) to control for protein loading.

### Analysis of drug interaction

To characterize the pharmacological impact of adding mifepristone to the standard cisplatin-paclitaxel combination chemotherapy, we used the CalcuSyn software (Biosoft, Cambridge, UK). This program utilizes the combination index (CI) as a method for quantifying drug cytotoxic synergism based on the mass-action law as designed by Chou and Talalay [23,24]. Synergism is defined as a more than expected additive effect, and antagonism is defined as a less than expected additive effect. Percent growth inhibition was used as a variable for the dose–response analyses, and the CI was calculated utilizing as ‘effect level’ or ‘fraction affected’ level the percent growth inhibition divided by 100. For drug interaction purposes, the combination cisplatin-paclitaxel was considered as one variable. Cells were exposed to various doses of cisplatin in the range of 2–20 μM, paclitaxel in the range of 0.5-100 nM, and mifepristone in the range of 5–20 μM. When combinations cisplatin-paclitaxel and cisplatin-paclitaxel-mifepristone were tested, cisplatin was fixed at 20 μM, mifepristone at 10 μM, and paclitaxel was varied in the range of 0.5-100 nM. To the specified drug association, CI = 0.9-1.1 denotes an additive effect, CI = 0.7-0.9 indicates slight synergism, CI = 0.3-0.7 indicates strong synergism, whereas CI >1.1 indicates antagonism. Drug interaction among the combination cisplatin-paclitaxel with mifepristone for the different cell lines was expressed as normalized isobolograms for the most relevant combination data point leading to synergism. The median dose (Dm) of each single drug and the dose reduction index (DRI) were also calculated. Dm values indicate median-effect dose or concentration, which is usually depicted as IC_50_. DRI is a measure of how much the dose of each drug can be reduced to obtain any given biological effect when compared with the doses for each drug alone. Although a DRI > 1 is beneficial, it does not necessarily indicate synergism; however it is important from a clinical standpoint where dose-reduction predicts reduced toxicity toward the host while retaining therapeutic efficacy.

### Clonogenic survival assay

Twenty one days after challenge with the drugs, 500 viable cells were placed in 6-well plates and cultured for 7 days until colonies were large enough to be clearly discerned. At this point, the medium was aspirated; the dishes were washed twice with PBS, fixed with 100% methanol for 30 min, and stained with a filtered solution of 0.5% (w/v) crystal violet (Sigma) for 10 min. The wells were then washed with tap water and dried at room temperature. The colonies, defined as groups of ≥30 cells, were scored manually with the aid of a Nikon Diaphot inverted microscope (Nikon, Garden City, NY). Clonogenic survival was expressed as the number of colonies formed during the different treatment paradigms.

## Results

### Exposure of ovarian cancer cells to cisplatin and paclitaxel induces substantial growth inhibition and lethality that are either unaffected or enhanced by chronic presence of a cytostatic dose of mifepristone after removal of the cytotoxic agents

**Figure 1:**
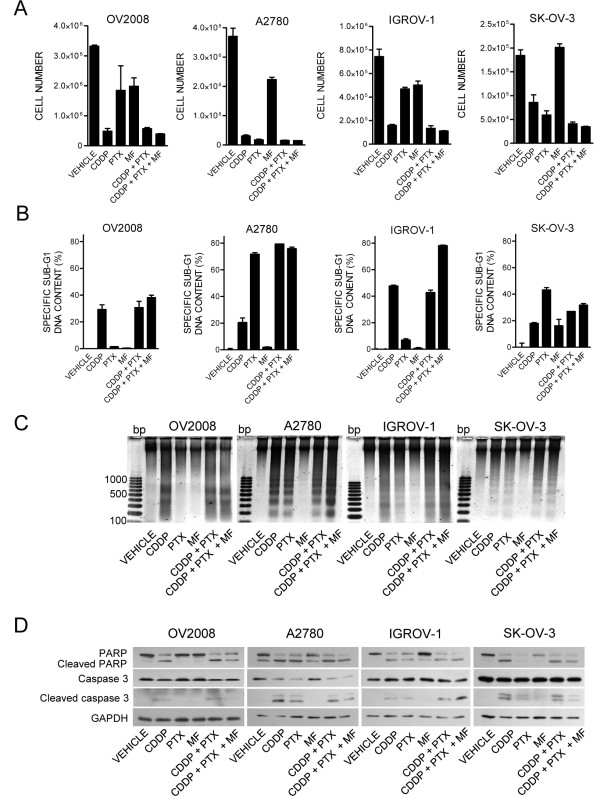
**Short-term cytotoxicity of cisplatin (CDDP) and paclitaxel (PTX) towards ovarian cancer cells is unchanged or enhanced by mifepristone (MF).(A)** Number of ovarian cancer cells measured 4 days after exposure to 20 μM CDDP for 1 h, 100 nM PTX for 3 h, 10 μM MF for 4 days, the combination of CDDP plus PTX, or CDDP plus PTX followed by MF. **(B)** Hypo-diploid DNA content calculated for the same experimental groups depicted in (A). **(C)** A similar experiment was done in which all floating and adherent cells were pelleted, total DNA isolated, subjected to agarose electrophoresis, stained with SYBR Gold nuclei acid stain, and photographed with the Amersham Typhoon Fluorescence imaging system. A 100 base pair (bp) marker was run in parallel. **(D)** In a similar experiment as in (C), whole protein extracts were obtained and separated by electrophoresis, and immunoblots were probed with the indicated antibodies. The housekeeping gene GAPDH was used as protein loading control

To study whether mifepristone is capable of improving the efficacy of cisplatin when the platinating agent is combined with paclitaxel, we set up a preclinical *in vitro* model system using exposure times and concentration ranges known to cause lethality [25,26]. We exposed ovarian cancer cells of different genetic backgrounds (OV2008, A2780, IGROV-1, and SK-OV-3) to 20 μM cisplatin for 1 h, and/or 100 nM paclitaxel for 3 h. After removal of the cytotoxic drugs, the cells were maintained in media with or without a cytostatic, 10 μM maintenance dose of mifepristone. The acute cytotoxicity of cisplatin, paclitaxel or the combination cisplatin-paclitaxel was evidenced by the reduction in cell density and increased lethality observed 4 days after treatment. In terms of inhibition of cell growth (Figure 1A), cisplatin alone reduced cell density remarkably in OV2008, A2780, and IGROV-1 cells, but was slightly less effective in SK-OV-3 cells. Paclitaxel was highly toxic to A2780 and SK-OV-3 cells, but less so to IGROV-1 and OV2008 cells. Mifepristone monotherapy, at the dose utilized, had a mild cytostatic effect in OV2008, A2780 and IGROV-1 cells, but no evident effect on SK-OV-3 cells. Cells treated with cisplatin and paclitaxel were largely affected in their growth capacity regardless of their genetic backgrounds, thus confirming *in vitro* their combined efficacy shown in the clinic. The chronic presence of mifepristone following the acute exposure to cisplatin and paclitaxel did not interfere with the action of the chemotherapeutic drugs. Instead, in some cases, mifepristone seemed to enhance the effect of the combination cisplatin-paclitaxel (see below).

We next studied the killing capacity of the various treatment paradigms by assessing the hypo-diploid DNA content, DNA fragmentation, and caspase-3 activation, all known markers of cisplatin-paclitaxel-induced toxicity [4,27-33]. Cisplatin alone was lethal to all cell lines as assessed by hypodiploid DNA content (Figure 1B), fragmentation of the DNA (Figure 1C) and cleavage of executer of apoptosis, caspase-3, and of its downstream substrate PARP (Figure 1D). Paclitaxel alone induced lethality to all cell lines except to OV2008 cells (Figure 1B-D). As expected, based on our previous studies [12,17,19,20], cells receiving mifepristone monotherapy did not display any evidence of cellular damage at the concentrations used. The combination cisplatin-paclitaxel was lethal to all cell lines, irrespective of their genetic backgrounds (Figure 1B-D). The chronic presence of mifepristone following cisplatin-paclitaxel did not interfere with the toxicity caused by the standard drugs. On the contrary, for instance in IGROV-1 cells, the presence of mifepristone enhanced cisplatin-paclitaxel toxicity as reflected by specific sub-G1 DNA content (Figure 1B) and caspase-3 activation (Figure 1D). Collectively, results in Figure 1 confirm that in combination, cisplatin and paclitaxel are highly efficient in growth inhibiting and/or killing ovarian cancer cells regardless of their genetic makeup, and demonstrate that a follow-up, chronic, non-toxic dose of mifepristone does not interfere with the primary toxicity of cisplatin-paclitaxel.

### Time-course of cell growth and cell death-related events occurring following cytotoxic cisplatin-paclitaxel therapy in cells subjected or not to the chronic presence of mifepristone

**Figure 2:**
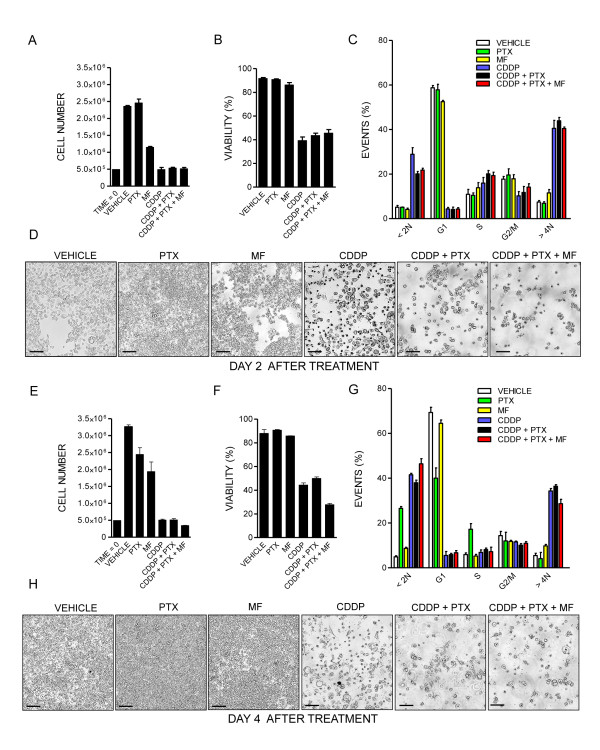
**Time-course kinetics of cytotoxicity triggered by cisplatin (CDDP) and paclitaxel (PTX) followed or not followed by mifepristone (MF) in OV2008 cells.** Number of cells **(A** and **E),** viability **(B** and **F)**, distribution within the cell cycle **(C** and **G)** and phase contrast images (**D** and **H**) were obtained 2 days (A through D) or 4 days (E through H) following initial exposure to CDDP, PTX, MF, the combination CDDP-PTX, or the triplet CDDP-PTX-MF. Scale bar, 120 μm.

We utilized OV2008 to study in further detail the kinetics of the effect of cisplatin, paclitaxel, mifepristone or their combination. We treated cells with cisplatin for 1 h, paclitaxel for 3 h, then removed the drugs, and exposed the cultures to fresh media containing or not containing mifepristone. We assessed cell number, viability, cell cycle distribution and morphology of the culture on days 2 and 4 following treatment. During the experiment we did not replace the media in order to assess the fate of the total cellular mass, thus documenting the overall process of toxicity associated with the simultaneous presence of adherent and non-adherent cells; we have evidence that mifepristone has a long lasting effect in culture (data not shown). Cells receiving only vehicle grew significantly from day 2 to day 4, had a healthy morphology and over 90% viability with cells distributed among the different phases of the cell cycle (Figure 2A-H). As anticipated in previous experiments, paclitaxel alone slightly affected cell growth by day 4 and caused a small increase in sub-G1 DNA content, yet without detriment of viability. Cells receiving cisplatin alone did not grow, declined their viability and displayed morphology with major cellular damage, and DNA content with abundant hypodiploidism and hyperploidism. Mifepristone alone was mildly cytostatic and did not cause either loss of viability or increase in hypodiploid DNA content. Cells receiving the combination paclitaxel-cisplatin showed reduced number, reduced viability, and signs of extended toxicity in their morphology and at the level of DNA distribution, having cells with hypodiploidism or hyperploidism. The triplet cisplatin-paclitaxel-mifepristone displayed the largest toxicity, with the maximal reduction in viability, and increase in sub-diploid DNA content, while preserving the hyperploidism also observed in cells receiving cisplatin alone or cisplatin plus paclitaxel. For all the groups studied respectively on days 2 and 4, Additional file 1: Figures S1 and Additional file 2: Figure S2 show the histograms of the viability (panel A) and DNA distribution (panel B) as measured by flow cytometry.

### Cisplatin-paclitaxel combination therapy is efficient in the short term causing substantial cellular damage, yet culture repopulation ensues with time; such repopulation can be prevented by the presence of mifepristone

**Figure 3:**
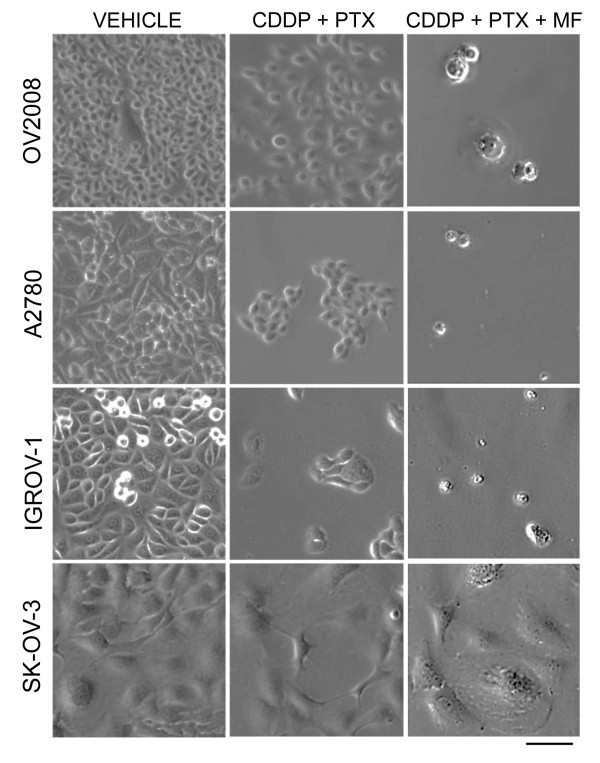
**Ovarian cancer cells escaping cisplatin (CDDP)-paclitaxel (PTX) therapy repopulate the culture, a phenomenon that is limited by the chronic presence of mifepristone (MF).** Depicted are images obtained by phase contrast microscopy 7 days after initial exposure to vehicle, CDDP-PTX, of the triplet CDDP-PTX followed by chronic presence of MF. Scale bar, 200 μm.

OV2008, A2780, IGROV-1 and SK-OV-3 cells treated with 20 μM cisplatin and 100 nM paclitaxel for 1 h and 3 h respectively, despite the apparent efficacy of the treatment in terms of cytotoxicity within the first 4 days following drug removal (Figures 1 and 2), escaped, eventually recurred, and repopulated the culture plate. We documented the escape/repopulation phenomenon following cytotoxic therapy in OV2008, A2780, IGROV-1 and SK-OV-3 cell lines. The cell cultures were photographed on day 7 following cisplatin-paclitaxel or cisplatin-paclitaxel followed by chronic exposure to mifepristone (Figure 3). Repopulating escape cells after cisplatin-paclitaxel show similar morphologies than untreated cells in their exponential phase of growth (Figure 3, middle and left panels). However, when 10 μM mifepristone was present in the culture media following the removal of the cytotoxic drugs, almost no colonies were observed in OV2008, A2780 and IGROV-1 cultures, while the anti-repopulation efficacy of mifepristone was only modest in SK-OV-3 cells, which nonetheless displayed enlarged morphology (Figure 3, right panel).

**Figure 4:**
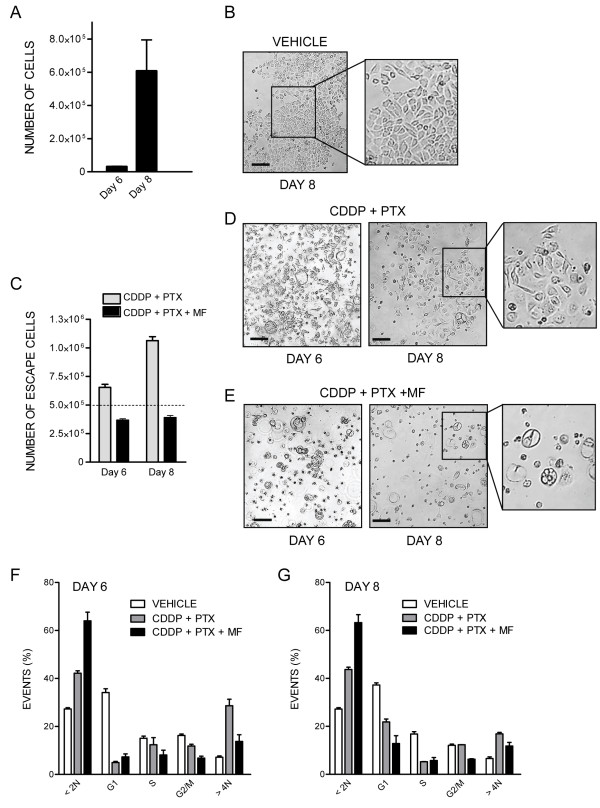
**Mifepristone (MF) abrogates the formation of escape foci of OV2008 cells following cisplatin (CDDP)-paclitaxel (PTX) therapy.** Number **(A)** and morphological features **(B)** of OV2008 cells in exponentially growing, untreated cultures 6 or 8 days after plating. **(C)**, **(D)**, and **(E)** depict the number and morphological features of cells that were exposed to CDDP and PTX and then either maintained untreated or exposed to MF for 6 or 8 days. In **(C)** the dashed line represents the number of cells initially plated. Panels **(F)** and **(G)** depict the DNA content of exponentially growing cells (VEHICLE), cells that received CDDP and PTX, or cells exposed to CDDP-PTX plus MF as measured on day 6 (**F**) or 8 (**G**) after initial treatment. Scale bars, 120 μm.

We analysed the phenomenon of repopulation in further detail in OV2008 that were observed on days 6 and 8 following initial treatment with cisplatin-paclitaxel, or cisplatin-paclitaxel followed by mifepristone. For this experiment we did not replace the culture media for the 8 days the experiment lasted in order to document all cellular events taking place. Due to the long term culture, as controls, we used cells that were plated at lower density to avoid growth arrest due to cell contact inhibition. Figure 4A shows that untreated cells, despite the lack of media change, were able to significantly growth, and to display an overall healthy morphology (Figure 4B), although an increase in hypodiploid DNA content consistent with a certain degree of cellular damage can be measured (Figure 4F). Cultures receiving cisplatin plus paclitaxel had an increased cellular density when compared to the culture of origin (Figure 4C). The culture shows coexistence of pockets of cells with apparent normal morphology emerging among heavily damaged cells (Figure 4D). This damage is also reflected in the elevated hypodiploid DNA content of cells receiving cisplatin-paclitaxel and studied 6 or 8 days after initial treatment (Figure 4F and G). Yet a population of cells pre-treated with cisplatin-paclitaxel is entering the cell cycle as reflected by the increase in the percentage of cellular particles allocated to the G1 phase on day 8 (Figure 4G) when compared to the same culture 2 days earlier (Figure 4F). Cultures receiving cisplatin-paclitaxel followed by chronic exposure to 10 μM mifepristone, in contrast, show reduced number of cells on day 8 following cisplatin-paclitaxel exposure, when compared to the number of cells originally plated (Figure 4C). The culture displays more than 60% of particles with hypo-diploid DNA content consistent with apoptosis (Figure 4F), and there is coexistence of large, heavily vacuolated cells with small and likely dead cells, without the presence of healthy pockets of repopulating cells (Figure 4E). The effect of mifepristone was further emphasized when used at a higher concentration following paclitaxel-cisplatin (Additional file 3: Figure S3).

In summary, data presented in Figures 1–4 suggest that despite cisplatin alone or in combination with paclitaxel is/are efficient in killing ovarian cancer cells, there are cells that escape the cytotoxic challenge. These escape cells do not die. Instead they are capable of repopulating the culture plate. This phenomenon, nonetheless, is abrogated by the presence of mifepristone.

### The blockage of repopulation by mifepristone of cells escaping cisplatin-paclitaxel is synergistic from a pharmacological standpoint

To quantify the added efficacy of mifepristone when blocking cell repopulation following cytotoxic cisplatin-paclitaxel therapy, we evaluated the growth inhibition properties of the drugs 7 days following the acute challenge with cisplatin for 1 h, paclitaxel for 3 h, cisplatin for 1 h and paclitaxel for 3 h, or the combination of acute cisplatin-paclitaxel challenge followed or not by chronic exposure to 10 μM mifepristone. Single agent IC_50s_ (concentrations needed to inhibit cellular growth by 50%) ranged from 18.2 to more than 170 nM for paclitaxel, from 3.59 to more than 14 μM for cisplatin, and from 6.8 to 15.8 μM for mifepristone (Additional file 4: Table S1). Cisplatin was more cytotoxic to OV2008, A2780 and IGROV-1 cells, all considered platinum sensitive [22], when compared to SK-OV-3 cells that were obtained from a patient resistant to clinically achievable concentrations of cisplatin, and are considered semi-resistant *in vitro*[32]. In terms of paclitaxel response, OV2008 were less sensitive, whereas A2780, IGROV-1 and SK-OV-3 were more sensitive to concentrations lower than 100 nM, confirming previous reports [4,34-36]. All cell lines were growth inhibited by the cytostatic agent mifepristone, confirming our previous results [12,17,20].

**Figure 5:**
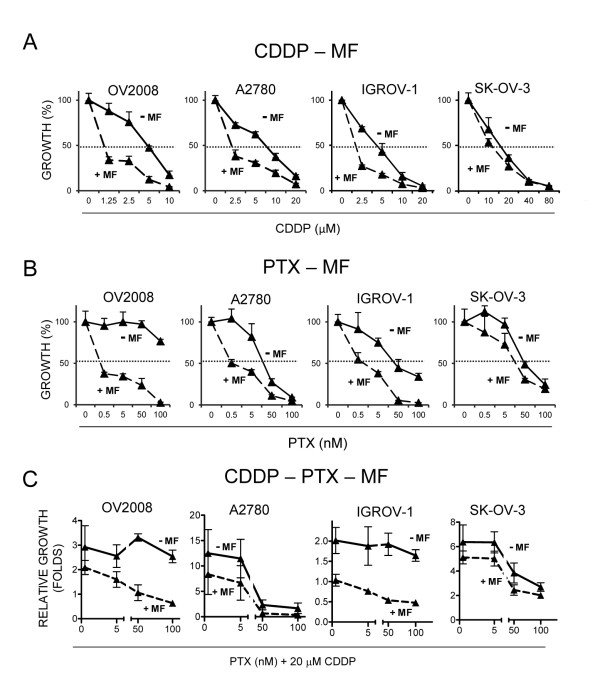
**Mifepristone (MF) enhances the therapeutic efficacy of cisplatin (CDDP) and paclitaxel (PTX) as shown by comparative dose response curves.** Cells were treated with CDDP for 1 h (**A** and **C**), PTX for 3 h (**B** and **C**), and exposed or not exposed to 10 μM MF for 7 days (**A-C**). Cell growth (**A** and **B**) was calculated considering controls as 100%. In (**C**), relative growth was assessed by considering 1 the number of cells at the beginning of the experiment. The dashed lines represent 50% of the growth depicted by control, untreated cells after 7 days of incubation.

To study whether the presence of mifepristone potentiated the therapeutic efficacy of cisplatin, paclitaxel or the combination of cisplatin-paclitaxel, we studied cell growth in the presence of increasing concentrations of cisplatin (Figure 5A), paclitaxel (Figure 5B) or the combination of a fixed dose of cisplatin with varying doses of paclitaxel (Figure 5C). Parallely, we cultured cells with similar doses of cisplatin and paclitaxel but adding a fixed, 10 μM concentration of mifepristone to the culture media. Data show that presence of mifepristone decreased the concentration of cisplatin needed to achieve the IC_50_ (shown by a dashed line) in OV2008, A2780 and IGROV-1, but not in SK-OV-3 cells (Figure 5A). Adding mifepristone to cells cultured with varying doses of paclitaxel reduced largely the IC_50s_ also in OV2008, A2780, and IGROV-1, but not in SK-OV-3 cells (Figure 5B). Finally, when mifepristone was added to a fixed concentration of cisplatin and varying doses of paclitaxel, the potentiation of growth inhibition induced by the presence of mifepristone was clearly observed in OV2008 and IGROV-1 cells, but less so in A2780 and SK-OV-3 cells (Figure 5C).

**Figure 6:**
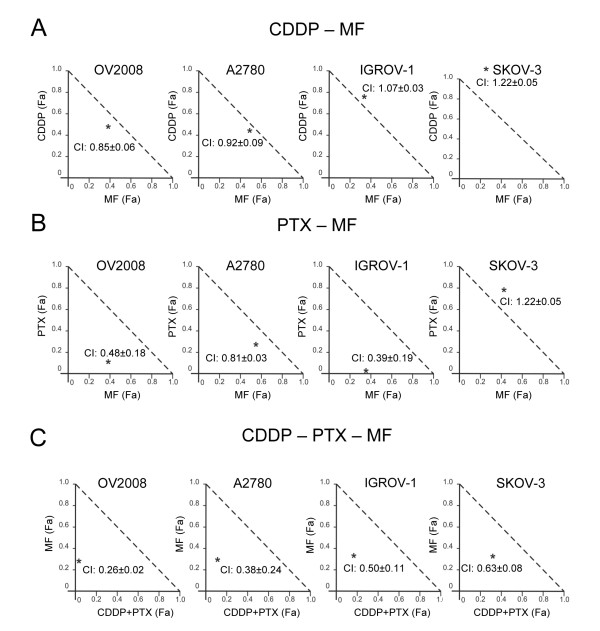
**Normalized isobolograms depicting the pharmacological interaction of mifepristone (MF) when it follows cisplatin (CDDP) and/or paclitaxel (PTX) as calculated from the comparative dose response curves depicted in** Figure 5**.** (**A**) Interaction between CDDP and MF. (**B**) Interaction among PTX and MF. (**C**) Interaction between MF and the combination CDDP-PTX, which was considered as ‘one treatment’ for the calculation purposes. The normalized isobolograms were obtained using CalcuSyn software. The depicted combination indexes (CI) in the isobolograms (*) were calculated using the doses of 20 μM CDDP, 100 nM PTX and 10 μM MF; n = 3.

To determine whether the nature of the potentiation induced in some of the ovarian cancer cell lines by mifepristone over cisplatin, paclitaxel, or the combination cisplatin-paclitaxel was additive or synergistic, we assessed the degree of interaction among clinically relevant doses of 10 μM mifepristone, and lethal concentrations of cisplatin (20 μM) and paclitaxel (100 nM), utilizing the drug combination algorithm of Chou and Talalay [23,24]. The interaction between mifepristone and cisplatin was synergistic in OV2008 and A2780 cells, additive in IGROV-1 cells, and slightly antagonistic in SK-OV-3 cells as shown by the normalized isobolograms displayed in Figure 6A. The interaction among mifepristone and paclitaxel was synergistic in OV2008, A2780 and IGROV-1 cells, but slightly antagonist in SK-OV-3 cells (Figure 6B). Finally, we assessed drug interaction when mifepristone was added to the combination cisplatin-paclitaxel. In this context, the mixture cisplatin-paclitaxel behaves as third drug to the cells and, consequently, from the mathematical standpoint it was considered as just one treatment or drug. Addition of mifepristone to the cisplatin-paclitaxel mixture had a therapeutic advantage in terms of growth inhibition in all cell lines studied as suggested by combination indexes in the range of synergism (CI = 0.3–0.7) or strong synergism (CI = 0.1–0.3) (Figure 6C). The therapeutic advantage of adding mifepristone to the cisplatin-paclitaxel chemotherapeutic schedule is further reinforced by the calculation of the dose reduction index (DRI). Additional file 5: Table S2 shows the DRI that indicate how many folds the dose of cisplatin, paclitaxel, or mifepristone, within the scenario of a synergistic combination, may be reduced at a given effect level compared with the dose of each drug alone, and in order to achieve the IC_50_. The DRI were positive for cisplatin when combined with mifepristone or with paclitaxel/mifepristone, for paclitaxel when combined with mifepristone or cisplatin-mifepristone, and for mifepristone, when combined with paclitaxel or paclitaxel-mifepristone. All DRI > 1 are relevant from a clinical perspective because dose-reduction suggest that similar efficacy with lower toxicity may be achieved.

### Mifepristone blocks time-dependent repopulation and abolishes clonogenic survival of ovarian cancer cells that escape cisplatin-paclitaxel cytotoxic therapy

**Figure 7:**
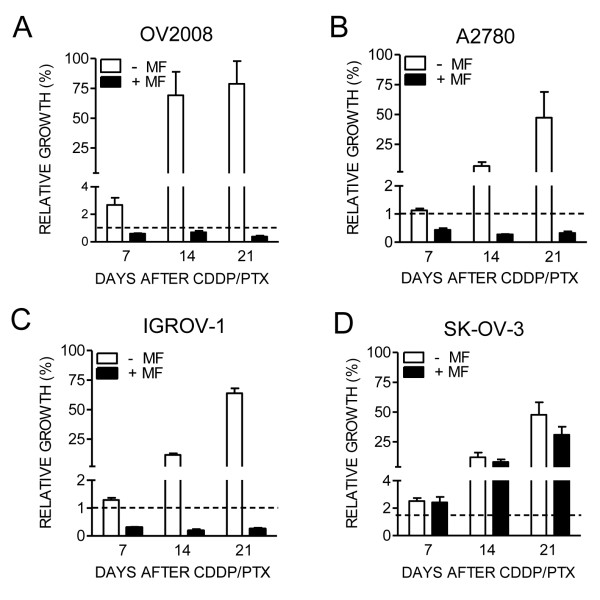
**The long-term repopulation of escape cells following cisplatin (CDDP)-paclitaxel (PTX) cytotoxic therapy is abrogated or limited by mifepristone (MF) in OV2008 (A), A2780 (B), IGROV-1 (C) and SK-OV-3 (D) cells.** Cells were exposed to 20 μM CDDP for 1 h, 100 nM PTX for 3 h and either chronically exposed or not exposed to 10 μM MF. Number of cells was assessed after one, two or three weeks of initial treatment. The dashed lines represent the relative number of cells originally plated before subjecting the culture to the various treatment schedules.

The regrowth of cells escaping cisplatin-paclitaxel toxicity was time-dependent with a marked increase in culture density as measured on days 7, 14 or 21 following initial chemotherapy (Figure 7). However, chronic exposure to a low cytostatic concentration of mifepristone blunted such cellular escape in all days studied. Cultures of OV2008, A2780 and IGROV-1 cells exposed to mifepristone following cisplatin-paclitaxel show a cellular density lower than the cellular density originally plated (Figure 7, dashed line). Mifepristone, however, was less efficacious in preventing escape of SK-OV-3 cells.

**Figure 8:**
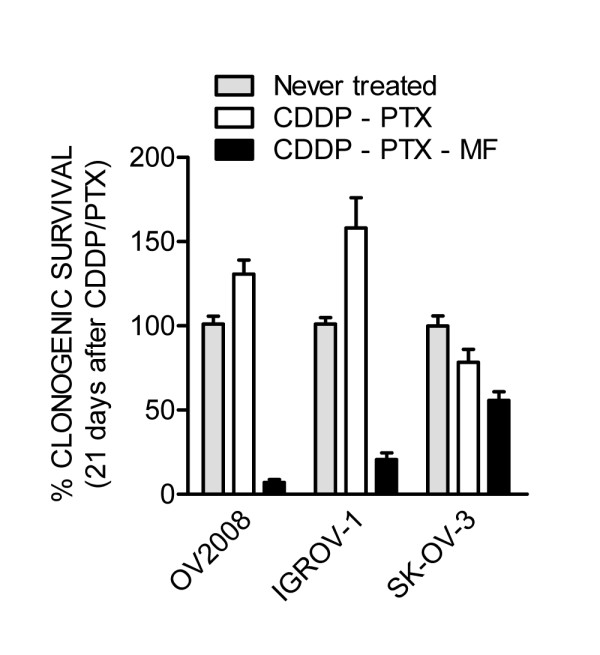
**Mifepristone (MF) diminishes the clonogenic survival of escape cells repopulating after cisplatin (CDDP)-paclitaxel (PTX) cytotoxicity.** Viable cells from a control culture, or from previous experiment as collected on day 21 after initial treatment, were subjected to a clonogenic survival assay. The number of positive colonies formed after one week of plating was assessed and the data were expressed relative to the clonogenicity elicited by untreated cells.

To determine whether there is an added long-term lethal imprint by mifepristone in cells pre-treated with cisplatin-paclitaxel, we subjected OV2008, IGROV-1 and SK-OV-3 cells that remained viable 21 days after treatment initiation, to a clonogenic survival assay. Results shown in Figure 8 reveal that escape cells following cisplatin-paclitaxel therapy have a growth advantage when compared to the cells of origin in the OV2008 and IGROV-1 cell lines. SK-OV-3, to which mifepristone did not appear to offer much advantage in terms of blocking repopulation assessed as cellularity in culture (Figure 7), showed a slightly reduced clonogenic survival in cells primed with cisplatin-paclitaxel, and a much more reduced clonogenic capacity in cells primed with cisplatin-paclitaxel but followed by chronic mifepristone maintenance therapy. The A2780 cells that escaped cisplatin-paclitaxel toxicity were not able to be studied in a clonogenic survival assay as they behave differently than the original A2780 cells, showing a live population of cells arranged in clusters that survive without adherence, thus invalidating the nature of the clonogenic survival assay that is based on counting adherent colonies (data not shown). When however total number of cells (either adherent of non-adherent) where counted in the clonogenic plate, the number of escape A2780 cells originally treated with cisplatin-paclitaxel and followed by mifepristone was highly reduced when compared to the number of cells escaping cisplatin-paclitaxel (data not shown), further confirming the phenomenon observed in the other cell lines in which mifepristone not only prevented escape of cells following cisplatin-paclitaxel therapy, but also impinged a long-term lethal influence.

## Discussion

Ovarian cancer is known as a silent killer due to its late detection and high mortality. Despite that much research has been done to improve the quality and quantity of life of ovarian cancer patients, the overall survival for this disease has remained stagnant at 30% since the introduction of the dual cisplatin-paclitaxel therapy in the mid-1990s. Efforts are geared at finding manners to diagnose the disease early, discovering target therapies, and improving the efficacy of current chemotherapeutic agents. We here provide evidence that when ovarian cancer cells are subjected to clinically relevant exposure times of cisplatin and paclitaxel, and to supra-pharmacological doses of the drugs –i.e. doses exceeding the maximal plasma concentrations that limit their tolerability, reported to be in the range of 50–100 nM for paclitaxel, and of 6–10 μM for cisplatin [37-40]—the therapy is initially successful, yet there are cells that eventually escape toxicity and relapse. Such relapse after remission can be, however, efficiently prevented by adding clinically relevant concentrations of the synthetic steroid mifepristone [13-16].

We modelled *in vitro* a scenario in which we utilized supra-pharmacological doses of cisplatin and paclitaxel [25,26] to maximize the cytotoxicity of the drugs, but limited their effect to clinically relevant times. Thus, cells were exposed to cisplatin in the range of 0–20 μM for only 1 h, to paclitaxel in the range of 0–100 nM for only 3 h, and chronically to 10 μM mifepristone, likely to be maintained *in vivo*[13-16]. Using this *in vitro* approach, we demonstrate that although the combination cisplatin-paclitaxel used at high doses is very efficacious in killing a large proportion of ovarian cancer cells encompassing broad genetic backgrounds, there are cells that escape the therapy and repopulate the culture following an otherwise apparent successful chemotherapeutic round.

The initial efficacy of the therapy with cisplatin and paclitaxel for ovarian cancer was amply validated and translated to the clinic many years ago [8,9]. However, the molecular mechanisms whereby cisplatin and paclitaxel alone or in combination cause cell toxicity necessitate further elucidation. Cisplatin displays cytotoxicity targeting the cytoplasm and the nucleus. In the cytoplasm, cisplatin interacts with a wide number of substrates tilting the redox balance to oxidative stress, which facilitates DNA damage [41]. Cisplatin also causes direct mitochondrial dysfunction [42] and endoplasmic reticulum stress [43]. In the nucleus, cisplatin binds DNA leading to the generation of DNA-DNA inter- and intra-strand adducts. These lesions cause distortions in the DNA that can be recognized by multiple repair pathways. When the extent of damage is limited, cisplatin adducts induce an arrest in the S and G2 phases of the cell cycle to allow the DNA repair mechanisms to re-establish DNA integrity and prevent abortive or abnormal mitosis. In contrast, if DNA damage is beyond repair, cells embark into a delayed death pathway [41,44-49]. Paclitaxel, on the other hand, acts by binding to intracellular β-tubulin leading to microtubule stabilization and G2-M arrest; thereafter, the cells may either die by apoptosis or necrosis immediately after the mitotic arrest, or following an aberrant mitotic exit into a G1-like multinucleated state [50]. Paclitaxel-induced cell death is, at least in part, mediated through the degradation of Bcl-2 [51]. In contrast to cisplatin, sensitivity to paclitaxel is independent of the p53 tissue expression status [52,53], while cisplatin-resistant cells retain sensitivity to paclitaxel [27,36]. There is no doubt of the initial efficacy of the combination cisplatin-paclitaxel; their synergistic pharmacological interaction has been amply proven [28,29]. In our work we substantiated that adding mifepristone after cisplatin-paclitaxel does not interfere with the efficacy of the dual combination, but rather, in some cases enhances it.

Dual cisplatin-paclitaxel therapy is followed by disease remission; however, it rarely provides cure as the disease eventually relapses after remaining dormant as minimal residual for over a year [6]. Such therapeutic failure is recapitulated in our *in vitro* toxicity model system in which scarce, yet critical cells escape cisplatin-paclitaxel therapy and regrow as demonstrated by their increase in number and by having a clonogenic survival capacity even superior than that of cells never treated with cisplatin-paclitaxel. When mifepristone was added after the initial toxic cisplatin-paclitaxel combination, repopulation was prevented and the clonogenic capacity of the remaining cells in culture was minimal.

The repopulation of cancer cells that escape or relapse after chemo- or radiotherapy accounts for the lack of long-term success of current cancer therapy. Repopulation of tumor cells was defined in 2005 by Kim and Tannock [54] as ‘the continuing proliferation, sometimes at an accelerated pace, of surviving tumor cells with the capacity to regenerate the tumor that can occur during a course of chemotherapy or fractionated radiotherapy.’ The mechanism of repopulation of escape cells, however, is less understood: some recent studies provide insights into it. For instance, cancer cells develop the capacity to escape DNA damage caused by pharmacological doses of platinum-based therapy via reverse polyploidy (a.k.a ‘neosis’ [55-57]), leading to the formation of diploid, rapidly proliferating cells with increased platinum resistance [58]. Such diploid descendants are formed upon reactivation of meiosis-specific genes from a polyploid genome [59,60], and in association with the formation of sub-nuclei that become degraded by autophagy [61]. Thus, a possibility exists that mifepristone blocks repopulation of escape cells by preventing reverse polyploidy. Supporting this hypothesis, we observed that OV2008 cultures that retain certain viability in between courses of cisplatin exposure, show giant cells together with a nascent population of small cells [62] that may originate from the likely polyploid, giant progenitors. Cultures treated with mifepristone after cisplatin do not show this small pool of repopulating cells; instead, they display an overall reduced number of cells, with predominance of a giant phenotype that ends up committing suicide as marked by cleaved PARP positivity [62]. Similar mechanism may be taking place in cells repopulating after cisplatin-paclitaxel combination therapy, because it is known that the driving force behind this therapy is cisplatin, not paclitaxel [44]. Additionally, within cultures repopulating after cisplatin-paclitaxel we observed a population of cells with hyperploid DNA content that is reduced parallel to cell repopulation and increased percentage of G1 cells. In cultured treated with cisplatin-paclitaxel plus mifepristone, however, such hyperploid population disappears in favor of hypodiploid DNA content consisting with cells dying by apoptosis instead of returning to the cell cycle.

Two other survival mechanisms that may explain repopulation after escape to chemotherapy could be the target of mifepristone interference. Firstly, mifepristone can block the release of survival factors from dying cells, because a recent study demonstrated that cells that are dying as a consequence of the chemotherapy, release chemical mediators (prostaglandins) that promote the growth of still surviving cells; more importantly, this mechanism requires caspase-3 activity in the dying cells [63], suggesting that caspase-3 has paradoxical functions, on one hand driving apoptotic cell death and on the other promoting the release of survival factors [64]. Secondly, there is also a possibility that mifepristone blocks the growth of scarce tumor initiating cells with the capacity to regenerate the culture and that may remain in culture because are resistant to cisplatin-paclitaxel in contrast to the bulk of differentiated cancer cells that succumb to the chemotherapy [65]. A genetic evolution study of high-grade serous ovarian adenocarcinomas suggests that resistance to cisplatin may develop from pre-existing minor clones that remain as minimal residual disease and become enriched after initial chemotherapy [66]. Within this scenario, mifepristone may block the repopulation of cells that never responded to cisplatin-paclitaxel therapy since, as we have shown, the drug has similar growth inhibition potency in platinum sensitive and platinum resistant ovarian cancer cells [20].

We provide proof-of-principle that the escape process following cytotoxic therapy can be abrogated by a chronic exposure to mifepristone. Long-term (months to years) of daily administration of mifepristone is feasible and clinically well tolerated [67]. Other synthetic steroid agents with similar structure than mifepristone containing a dimethylaminophenyl substitution at the 11-β position have been developed [68]. We demonstrated that, similar to mifepristone, the related steroids ORG-31710 and CDB-2914 block ovarian cancer cell growth in association with inhibition of the activity of cyclin dependent kinase 2 [17]. It warrants investigation whether these agents are equivalent or more efficient than mifepristone when used to block repopulation following cisplatin-paclitaxel therapy.

## Conclusions

Using an *in vitro* model of recurrence after exposure of ovarian cancer cells to supra-pharmacological doses of cisplatin and paclitaxel, and for clinically relevant exposure times, we demonstrated that a clinically relevant dose of the synthetic steroid mifepristone significantly improves treatment efficacy by reducing the number and clonogenic survival capacity of escape cells. Thus, mifepristone could be used for chronic, non-toxic maintenance therapy following cytotoxic standard cisplatin-paclitaxel chemotherapy to improve treatment efficacy by abrogating relapse of cells escaping cisplatin-paclitaxel.

## Competing interests

The authors declare that there is no conflict of interest that could influence the impartiality of the research reported.

## Authors' contributions

Conceived and designed the experiments: CGL AAG CMT. Performed experiments CGL AAG MBH. Analyzed the data: CGL AAG MBH CMT. Contributed reagents/materials/analysis tools: CMT. Wrote the paper: CGL CMT. All authors read and approved the final manuscript.

## Pre-publication history

The pre-publication history for this paper can be accessed here:

http://www.biomedcentral.com/1471-2407/12/200/prepub

## Supplementary Material

Additional file 1 **Figure S1.** Histograms representing the viability (upper panel) and DNA content (lower panel) of OV2008 cells assessed by microcytometric analysis 2 days following treatment with paclitaxel (PTX), mifepristone (MF), cisplatin (CDDP), CDDP-PTX, or the triplet CDDP-PTX-MF. FSC, forward scatter. Click here for file

Additional file 2 **Figure S2.** Histograms representing the viability (upper panel) and DNA content (lower panel) of OV2008 cells assessed by microcytometric analysis 4 days following treatment with paclitaxel (PTX), mifepristone (MF), cisplatin (CDDP), CDDP-PTX, or the triplet CDDP-PTX-MF. FSC, forward scatter. Click here for file

Additional file 3 **Figure S3.** Phase contrast images obtained from OV2008 cultures 4, 6, or 8 days following exposure to 20 μM cisplatin (CDDP) for 1 h, the doublet combination of 20 μM CDDP for 1 h and 100 nM paclitaxel (PTX) for 3 h, or the triplet combination of 20 μM CDDP for 1 h, 100 nM PTX for 3 h, and 20 μM mifepristone (MF) for the entire time in culture. Click here for file

Additional file 4: **Table S1.** Concentrations of cisplatin (CDDP), paclitaxel (PTX), or mifepristone (MF) that inhibit growth by 50% (IC_50s_) in ovarian cancer cells. Click here for file

Additional file 5: **Table S2.** Dose reduction index values (DRI) for cisplatin (CDDP), paclitaxel (PTX) and mifepristone (MF) in ovarian cancer cells. Click here for file
