# Supplementary figures and images for: Mifepristone prevents repopulation of ovarian cancer cells escaping cisplatin-paclitaxel therapy
Source: BMC Cancer. 2012 Jun 22;12:200. doi: 10.1186/1471-2407-12-200 (PMC3381704; doi:10.1186/1471-2407-12-200)

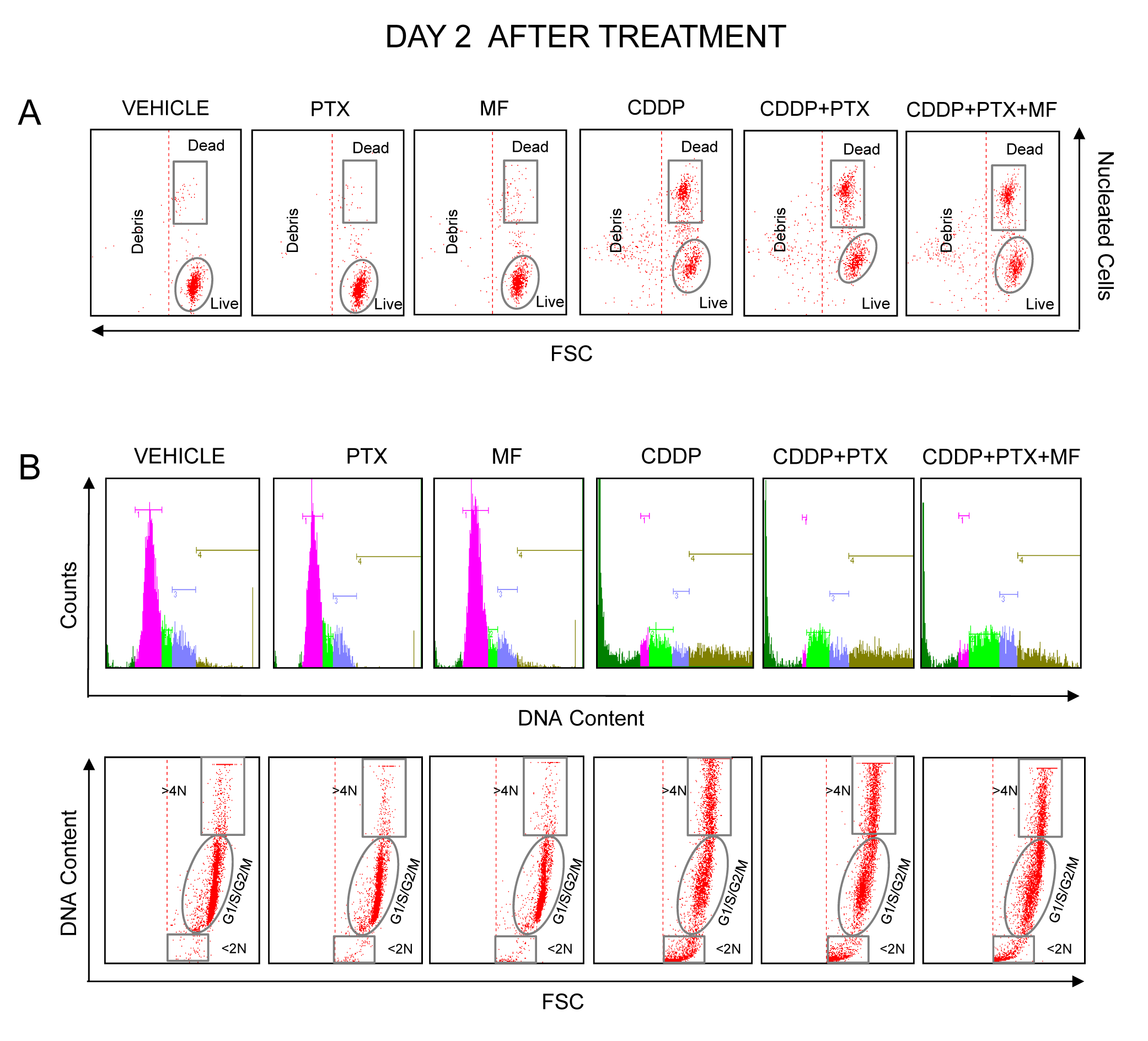

Supplement: Additional file 1 — Figure S1. Histograms representing the viability (upper panel) and DNA content (lower panel) of OV2008 cells assessed by microcytometric analysis 2 days following treatment with paclitaxel (PTX), mifepristone (MF), cisplatin (CDDP), CDDP-PTX, or the triplet CDDP-PTX-MF. FSC, forward scatter. [file 1471-2407-12-200-S1.tiff]

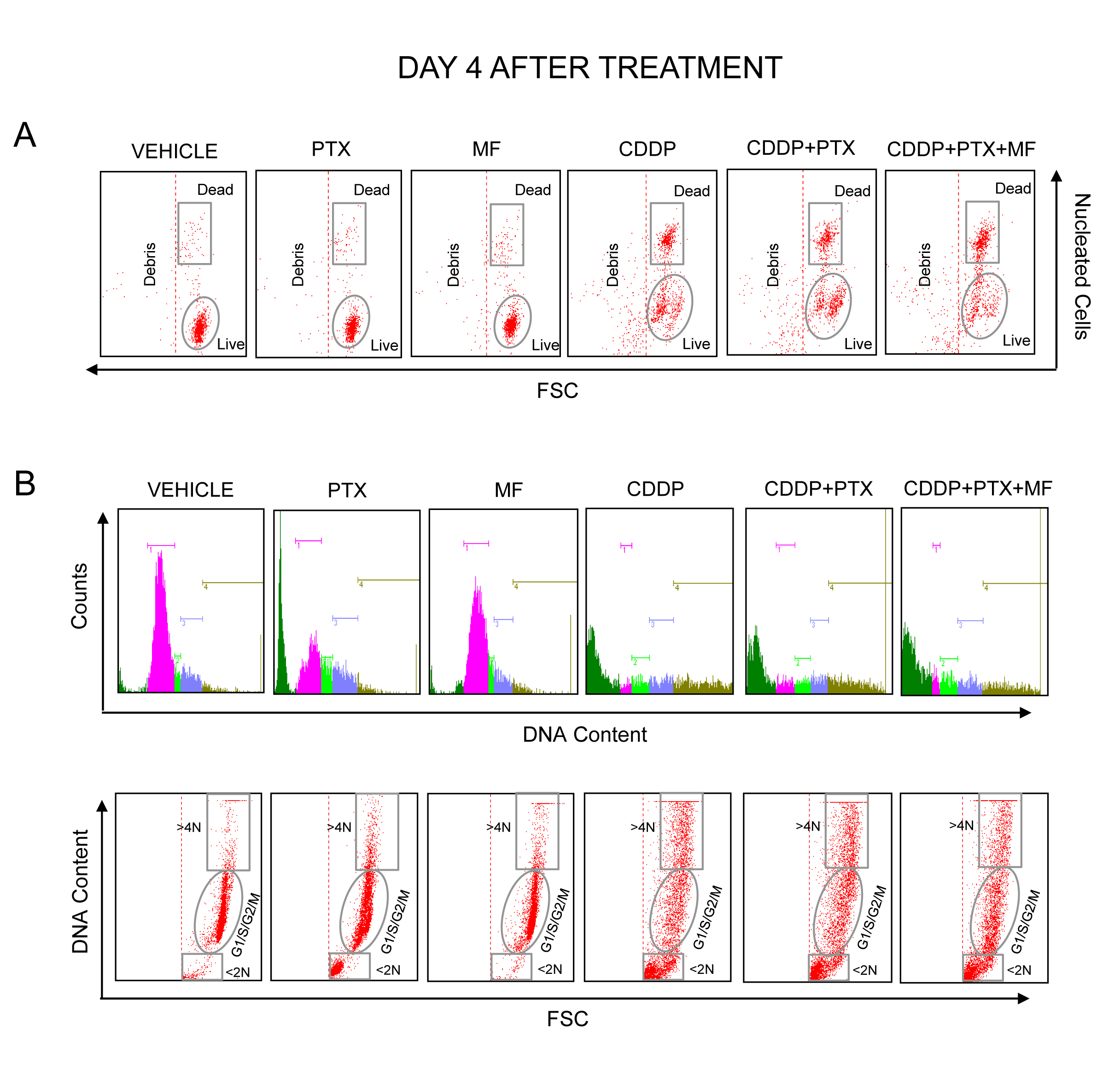

Supplement: Additional file 2 — Figure S2. Histograms representing the viability (upper panel) and DNA content (lower panel) of OV2008 cells assessed by microcytometric analysis 4 days following treatment with paclitaxel (PTX), mifepristone (MF), cisplatin (CDDP), CDDP-PTX, or the triplet CDDP-PTX-MF. FSC, forward scatter. [file 1471-2407-12-200-S2.tiff]

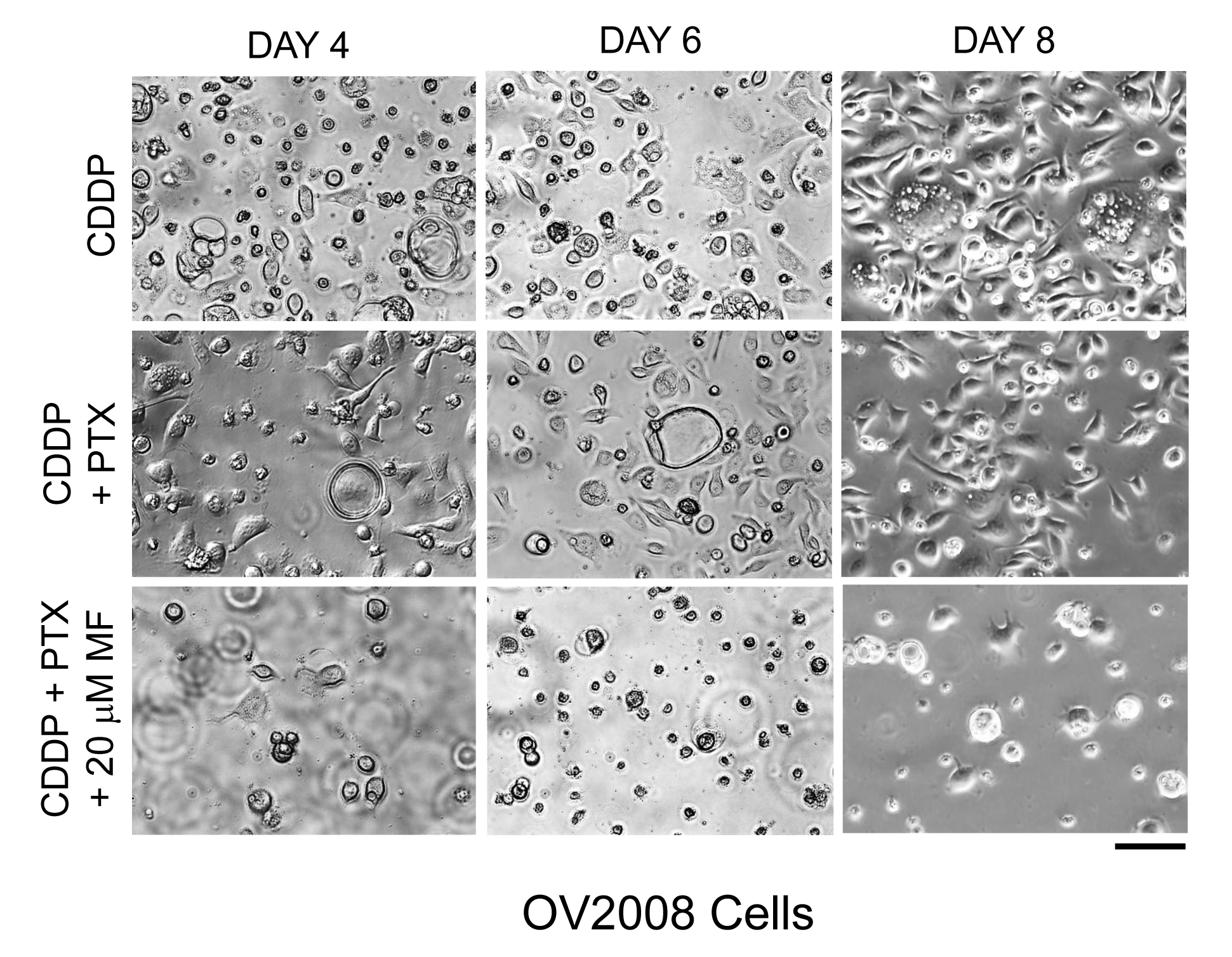

Supplement: Additional file 3 — Figure S3. Phase contrast images obtained from OV2008 cultures 4, 6, or 8 days following exposure to 20 μM cisplatin (CDDP) for 1 h, the doublet combination of 20 μM CDDP for 1 h and 100 nM paclitaxel (PTX) for 3 h, or the triplet combination of 20 μM CDDP for 1 h, 100 nM PTX for 3 h, and 20 μM mifepristone (MF) for the entire time in culture. [file 1471-2407-12-200-S3.tiff]
