# Supplementary material for: Mifepristone prevents repopulation of ovarian cancer cells escaping cisplatin-paclitaxel therapy
Source: BMC Cancer. 2012 Jun 22;12:200. doi: 10.1186/1471-2407-12-200 (PMC3381704; doi:10.1186/1471-2407-12-200)
Supplement: Additional file 4: — Table S1. Concentrations of cisplatin (CDDP), paclitaxel (PTX), or mifepristone (MF) that inhibit growth by 50% (IC50s) in ovarian cancer cells. [file 1471-2407-12-200-S4.docx]

**Table S1**

|  | **CDDP** | | | **PTX** | | | **MF** | | |
| --- | --- | --- | --- | --- | --- | --- | --- | --- | --- |
| **Cell line** | **IC_50_ (μM)** | ***r*** | **m** | **IC_50_ (nM)** | ***r*** | **m** | **IC_50_ (μM)** | ***r*** | **m** |
| **OV2008** | **3.59 ± 0.29** | **0.998 ± 0.003** | **1.99 ± 0.04** | **171 ± 20.7** | **0.998 ± 0.001** | **1.86 ± 0.09** | **6.80 ± 0.14** | **0.992 ± 0.005** | **2.61 ± 0.28** |
| **A2780** | **7.80 ± 2.27** | **0.995 ± 0.004** | **1.01 ± 0.07** | **18.2 ± 1.20** | **0.983 ± 0.002** | **1.13 ± 0.11** | **12.7 ± 1.15** | **0.984 ± 0.009** | **1.76 ± 0.21** |
| **IGROV-1** | **3.26 ± 0.51** | **0.993 ± 0.003** | **1.74 ± 0.14** | **31.3 ± 10.0** | **0.986 ± 0.014** | **0.57 ± 0.05** | **9.29 ± 0.24** | **0.996 ± 0.003** | **3.61 ± 0.20** |
| **SK-OV-3** | **14.5 ± 2.21** | **0.997 ± 0.001** | **1.71 ± 0.11** | **47.8 ± 3.90** | **0.993 ± 0.005** | **1.39 ± 0.10** | **15.7 ± 1.71** | **0.991 ± 0.005** | **2.74 ± 0.25** |

Cells were treated with CDDP for 1 h, PTX for 3 h, or MF for 7 days. All calculations were done assessing cell growth density on day 7. The experiment was repeated three times. IC_50_ are expressed as the mean ± SEM. *r* = the goodness-of-fit for the pooled data, where *r* = 1 represents a perfect fit. m = represents the sigmoidicity of the dose effect curve; m=1, >1, and <1 indicates hyperbolic, sigmoidal, and flat sigmoidal shape, respectively.
