# Supplementary material for: Mifepristone prevents repopulation of ovarian cancer cells escaping cisplatin-paclitaxel therapy
Source: BMC Cancer. 2012 Jun 22;12:200. doi: 10.1186/1471-2407-12-200 (PMC3381704; doi:10.1186/1471-2407-12-200)
Supplement: Additional file 5: — Table S2. Dose reduction index values (DRI) for cisplatin (CDDP), paclitaxel (PTX) and mifepristone (MF) in ovarian cancer cells. [file 1471-2407-12-200-S5.docx]

|  | **DRI for CDDP** | | | **DRI for PTX** | | | **DRI for MF** | | |
| --- | --- | --- | --- | --- | --- | --- | --- | --- | --- |
| **Cell line** | **+PTX** | **+MF** | **+PTX+MF** | **+CDDP** | **+MF** | **+CDDP+MF** | **+CDDP** | **+PTX** | **PTX+MF** |
| **OV2008** | **0.75 ± 0.10** | **2.54 ± 0.20** | **1.59 ± 0.11** | **9.69 ± 1.18** | **6.96 ± 0.65** | **8.87 ± 2.10** | **2.57 ± 0.29** | **2.98 ± 0.77** | **3.85 ± 1.30** |
| **A2780** | **3.57 ± 0.44** | **2.28 ± 0.22** | **5.89 ± 0.23** | **2.86 ± 1.14** | **2.02 ± 0.04** | **3.82 ± 0.66** | **2.06 ± 0.28** | **3.17 ± 061** | **4.03 ± 0.03** |
| **IGROV-1** | **1.27 ± 0.09** | **1.38 ± 0.09** | **2.37 ± 0.42** | **21.5 ± 4.00** | **183 ± 30.4** | **111 ± 5.25** | **2.92 ± 0.40** | **2.60 ± 0.55** | **3.36 ± 0.15** |
| **SK-OV-3** | **1.89 ± 0.27** | **1.27 ± 0.04** | **2.40 ± 0.68** | **2.10 ± 0.21** | **1.22 ± 0.28** | **2.74 ± 0.12** | **2.30 ± 0.44** | **2.48 ± 0.20** | **3.01 ± 0.58** |

**Table S2**

Cells were treated with CDDP for 1 h, PTX for 3 h, or MF for 7 days. All calculations were done assessing cell growth density on day 7. DRI=dose reduction index reflecting the fold reduction in the required concentration of tested agents when used in combination to achieve 50% growth inhibition. DRI are expressed as the mean ± SEM of three experiments performed each in triplicates.
